# Functional Characterization of the *PoWHY1* Gene from *Platycladus orientalis* and Its Role in Abiotic Stress Tolerance in Transgenic *Arabidopsis thaliana*

**DOI:** 10.3390/plants14020218

**Published:** 2025-01-14

**Authors:** Chun Ou, Zhiyu Dong, Xudong Zheng, Wenhui Cheng, Ermei Chang, Xiamei Yao

**Affiliations:** 1 Fuyang Normal University—Funan Rural Revitalization Collaborative Technology Service Center, School of Biology and Food Engineering, Fuyang Normal University, Fuyang 236037, China; dzy184379@163.com (Z.D.); 18055849995@163.com (X.Z.); cwh15293327149@163.com (W.C.); 2 Research Institute of Forestry, Chinese Academy of Forestry, Beijing 100091, China; 3 School of Architecture and Urban Planning, Anhui Jianzhu University, Hefei 230601, China

**Keywords:** *Platycladus orientalis*, *PoWHY1*, gene expression and analysis, abiotic stresses, drought

## Abstract

The frequent occurrence of extreme weather conditions in the world has brought many unfavorable factors to plant growth, causing the growth and development of plants to be hindered and even leading to plant death, with abiotic stress hindering the growth and metabolism of plants due to severe uncontrollability. The WHY1 transcription factor plays a critical role in regulating gene expression in plants, influencing chlorophyll biosynthesis, plant growth, and development, as well as responses to environmental stresses. The important role of the *PoWHY1* gene in regulating plant growth and adaptation to environmental stress has become a hot research topic. However, the mechanism of the *PoWHY1* gene in *Platycladus orientalis* under abiotic stress is still unclear. Here, the *PoWHY1* gene was analyzed bioinformatically using *P. orientalis* as study material, and the role of the gene against abiotic stress conditions in *Arabidopsis thaliana* was verified using transgenic technology. It was found that overexpression of *PoWHY1* increased seed germination, decreased malondialdehyde accumulation, increased proline content, and delayed the senescence process under salt stress. The expression levels of *JAZ1*, *LOX1*, *ABI1*, and *ABI2* were decreased, while the expression levels of *RAB18*, *APX1*, *GSTF6*, and *DREB2A* were increased, indicating that overexpression of *PoWHY1* enhanced the salt stress tolerance of *A. thaliana*. Furthermore, *PoWHY1* overexpression also increased drought tolerance in *A. thaliana*. From the above results, it can be concluded that maintaining high *PoWHY1* expression levels in the leaves of *P. orientalis* can improve their environmental adaptability. The results provide a scientific basis for understanding the gene function of the *PoWHY1* gene of *P. orientalis* under stress conditions and lay the foundation for further research on the function of the *PoWHY1* gene.

## 1. Introduction

Since the 21st century, extreme weather events have occurred frequently around the world, and adverse factors such as heavy rainfall, drought, typhoons, salinity, insect pests, and pathogen infections can cause plant growth retardation or even death, aggravating environmental degradation [1]. Among them, abiotic stresses such as drought, salinity, and temperature are the most important environmental factors that affect the geographical distribution of crops, limit crop yields, and threaten food security [2,3]. While biotic stresses are characterized by diversity, complexity, specificity, and controllability, they primarily affect plants through immune responses and protective mechanisms [4,5]. In contrast, abiotic stresses are generally uncontrollable and cause direct physical or chemical damage to plants. These stresses impair photosynthesis, nutrient absorption, and overall plant growth, disrupt cellular function, and can lead to excessive production of reactive oxygen species (ROS), which damage biomacromolecules such as cell membranes, proteins, and nucleic acids [6,7]. For example, when exposed to drought and osmotic stress, plants activate osmotic signaling through changes such as reduced swelling pressure, disruption of cell wall integrity, changes in membrane tension, cell damage, and macromolecular aggregation. These stress-induced changes are subsequently mediated by Ca^2+^ signaling or protein kinases, which mediate signaling at the plasma membrane to coordinate the plant’s response to stress [8,9]. Over the long process of natural selection, plants have also developed mechanisms to adapt to adverse environments. When plants are exposed to environmental stresses such as drought, high salinity, and low temperatures, hormone levels in the body change significantly, initiating or regulating the physiological and biochemical processes associated with stress tolerance. For example, in signaling in response to stress, abscisic acid (ABA) and jasmonic acid (JA) jointly regulate plant defense responses through complex interactions that are essential for coping with environmental and biotic stresses [10]. Plants also synthesize and accumulate a number of osmoregulatory substances such as proline, glycine betaine, etc., to maintain intracellular osmotic balance and water status and alleviate damage caused by drought and other stresses [8]. Plants can activate their antioxidant system to scavenge ROS in the body through antioxidants and enzymes to protect cells from oxidative damage, and they will also make adjustments to the structure of cells to improve their resilience [11]. In addition, plants adapt to environmental changes by regulating gene expression. Some genes related to stress tolerance are induced to synthesize more stress-resistant proteins and metabolites, while some genes related to normal growth and development are repressed to reduce energy expenditure and loss [12]. This regulation of gene expression is one of the important mechanisms for plant adaptation to abiotic stress.

*WHY* gene expression proteins are plant-specific transcription factors, characterized by their highly conserved single-stranded DNA-binding domains. These proteins are predominantly localized in the cell nucleus and various organelles, where they play a key role in the plant’s response to pathogenic bacterial attack, and stress, as well as growth and development. Additionally, WHY proteins contribute to the maintenance of DNA and telomere stability [13,14,15]. When a plant is exposed to abiotic stress in a localized tissue or organ, a systemic response is generated through intercellular signals to bring about general adaptation to the stress [5]. WHY proteins act as transcriptional activators of a large number of nuclear genes to participate in this highly sensitive and precise adaptive mechanism to cope with a variety of abiotic stresses in natural environments [16]. The WHY protein family is characterized by a typical secondary structure and a conserved KGKAAL DNA domain, which play crucial roles in single-stranded DNA binding and polymerization [17]. Most plants have two WHY proteins, WHY1 and WHY2, which differ in their organelle targeting [14]. WHY1 is a nuclear-localized protein that is also targeted to chloroplasts and plays a major role in pathogen-induced transcription, embryonic development, abiotic stress response, and genome repair [18,19]; WHY2 primarily targets mitochondria and exhibits triple localization to the nucleus, plastids, and mitochondria. In coordination with other single-stranded DNA-binding proteins, WHY2 plays a crucial role in maintaining mitochondrial DNA integrity and regulating organelle gene expression [20,21]. *WHY1* was initially identified in potato as a transcriptional activator capable of binding to the inducer element of the promoter of the pathogenic gene *PR-10a* [22]. It is also involved in cold stress resistance and the regulation of photosynthesis through its association with the *psbA* gene [15]. Subsequently, it was successively reported in a variety of plants. In barley, knockout of the *WHY1* gene delays leaf senescence under drought stress, a process mediated by the regulation of chromatin structure and gene expression [23]. Comadira found that mutant barley seedlings depleted of the *WHY1* gene had increased chlorophyll content under nitrogen deficiency stress and could still undergo photosynthetic CO_2_ assimilation. It can be concluded that under abiotic stress, *WHY1* plays a role in the communication between plastid and nuclear genes encoding photosynthetic proteins [24]. In tomatoes, Zhao found that overexpression of the tomato *WHY* gene in transgenic tobacco resulted in an increase in tobacco tolerance to drought stress and resistance to Pseudomonas syringae in tomatoes [25]. Zhuang found that *WHY1* maintains the photosynthetic capacity of tomato leaves by regulating the expression of RbcS1 under cold stress [26]. Additionally, WHY1 plays a crucial regulatory role in leaf senescence and can delay natural senescence through multiple mechanisms [27]. In *A. thaliana*, the single-stranded DNA-binding protein WHY1 delays leaf senescence by repressing *WRKY53* expression [28]. In barley, WHY1 functions as an upstream regulator of leaf senescence and binds to the promoters of senescence-associated genes (e.g., *HvS40*) to regulate the senescence process. It was also found that the expression of both senescence-associated genes and drought stress-responsive genes was delayed by knocking down the *HvWHY* using RNAi [29].

*P. orientalis* is an evergreen tree native to North America but widely distributed worldwide, with the advantages of drought and infertility resistance, disease resistance, and anti-aging properties. Due to its low water consumption, adaptability, etc., it is one of the most common ornamental species in China [30,31]. Owing to its exceptional stress tolerance, *P. orientalis* has been extensively utilized in landscape architecture, ecological restoration, construction materials, and essential oil extraction [32,33]. It also demonstrates significant potential for barren mountain reforestation and urban greening [34,35]. Moreover, ancient *platycypress* trees hold considerable scientific, cultural, and historical significance [36]. In recent years, *WHY1* genes, which are largely involved in plant stress response, have been identified and analyzed in many species. However, the identification and analysis of the *PoWHY1* gene family in *P. orientalis* is still unclear. In this study, leveraging the latest genomic data of *Platycladus orientalis*, we first investigated the expression patterns of *PoWHY1* under salt stress, heat stress, PEG6000-induced osmotic stress, and UV-C stress to gain a comprehensive understanding of its response to various abiotic stresses. Subsequently, an overexpression vector, *pCAMBIA1304-PoWHY1*, was constructed and introduced into *Arabidopsis thaliana* to examine the effects of salt and drought stresses on transgenic plants. This approach aimed to validate the functional role of *PoWHY1* in enhancing plant stress tolerance. Overall, this study seeks to elucidate the abiotic stress response mechanisms of *PoWHY1*, providing a scientific basis for further exploration of the *WHY* gene family and for improving plant stress resistance.

## 2. Results

### 2.1. Cloning of PoWHY1 Gene

The complete sequence of the *PoWHY1* gene was cloned from the RNA reverse transcription cDNA of 500-year-old *P. orientalis* leaves. Using the specific primers GSP2 and UPM, a 568 bp fragment was amplified by 3′ RACE-PCR. After 5′ RACE PCR amplification with specific primers GSP1 and UPM, a fragment with a length of 893 bp was obtained. According to the overlap region of the 5′ RACE and 3′ RACE amplification fragments, the complete sequence of the *P. orientalis PoWHY1* gene with a length of 1295 bp was finally obtained. The resulting full-length cDNA sequence was used to design primers at both ends of the ORF and perform PCR amplification to obtain the ORF of the *PoWHY1* gene (Figure S1).

### 2.2. Bioinformatics Analysis of PoWHY1 Genes

**Figure 1:**
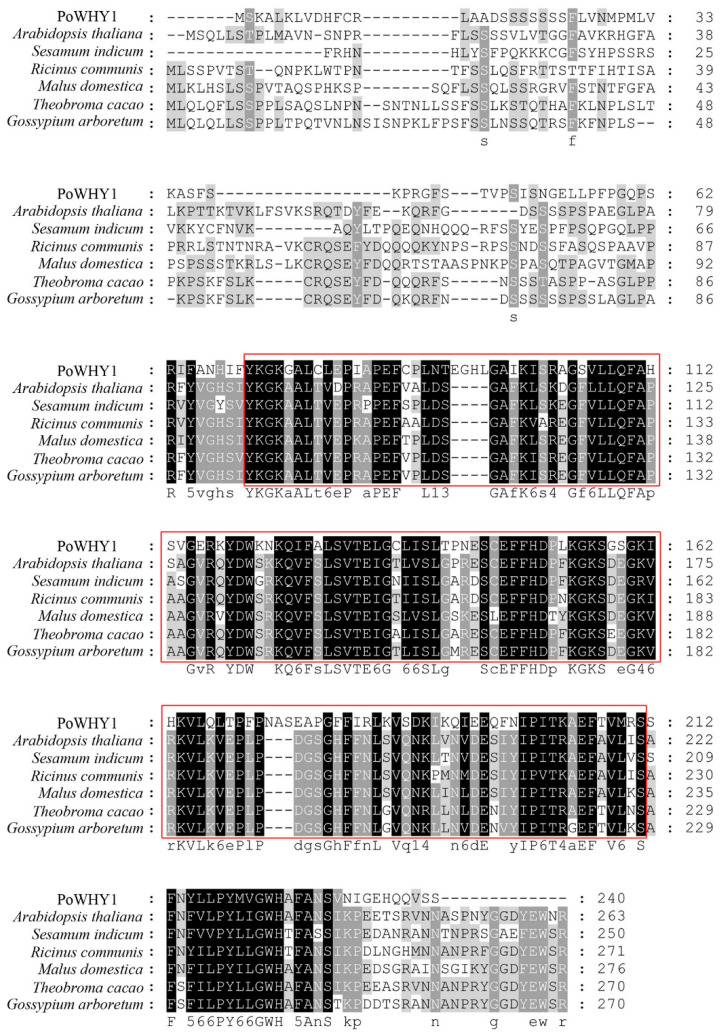
Alignment of the amino acid sequences of PoWHY1 and WHY1 from other plants. Whirly (71–211) domain is boxed.

**Figure 2:**
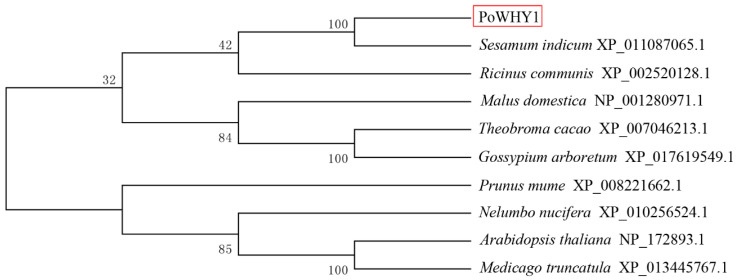
Phylogenetic relationship between PoWHY1 and WHY1 proteins from other plants. The portion highlighted with a red box is PoWHY1.

The *PoWHY1* gene was 1295 bp long, including 135 bp of 5′ non-coding region, 437 bp of 3′ non-coding region, 26 bp polyA tail, and 723 bp ORF. Protparam predicted that the *PoWHY1* protein contained 240 amino acids, of which 54.6% were polar amino acids, 45.4% were hydrophobic amino acids, 13.7% were basic amino acids, and 7.5% were acidic amino acids, and the molecular weight of the protein was estimated at 26.47 kDa. The theoretical isopotential was 9.19. SingalP prediction showed that *PoWHY1* had no signal peptide output site and was a non-secreted protein. Secondary structure prediction of *PoWHY1* by HNN software showed that PoWHY1 protein consisted of 35.83% α-helix (alpha-helix), 12.92% extended strand, and 51.25% random coil without β-corner. Therefore, random curl is a major component of the *PoWHY1* secondary structure. The *PoWHY1* protein contains a Whirly domain (pfam08536) located at positions 71–211 of the amino acid sequence (Figure 1). Subcellular localization by WoLF PSORT 0.2 software showed that the PoWHY1 protein had the highest probability of distribution in the nucleus. NCBI-BLAST search for PoWHY1 homologous amino acid sequences and alignment revealed that PoWHY1 shared some similarity with various plant WHY1 proteins in the GenBank database. The amino acid sequence of *PoWHY1* was 52%, 52%, 54%, 54%, 54%, 54%, and 56% similar to *A. thaliana* (NP_172893.1), *Sesamum indicum* (XP_011087065.1), *Ricinus communis* (XP_002520128.1), *Malus domestica* (NP_001280971.1), *Theobroma cacao* (XP_007046213.1), and *Gossypium arboretum* (XP_017619549.1). The phylogenetic tree (Figure 2) showed that *PoWHY1* had higher homology with *Sesamum indicum* than other species.

### 2.3. PoWHY1 Gene Expression Pattern Analysis

**Figure 3:**
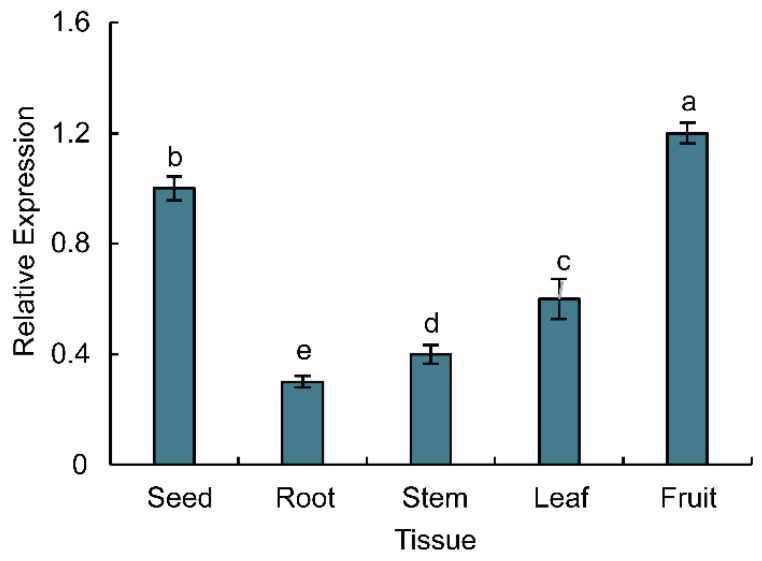
Expression patterns of *PoWHY1* in different tissues of *P. orientalis.* The letters a–e typically indicate statistical significance between groups. Groups with different letters show significant differences (*p* < 0.05).

The expression pattern of the *PoWHY1* gene in 20-year-old *P. orientalis* organs (seeds, roots, stems, leaves, and fruits) is shown in Figure 3. In the tissues of *P. orientalis*, the expression level of *PoWHY1* was higher in fruits, 1.13 times higher than in seeds, followed by seeds, and then decreased in leaves and stems, and the relative expression level in roots was the lowest, being 0.30 times as much as in seeds.

### 2.4. Abiotic Stress Response of PoWHY1 Gene

**Figure 4:**
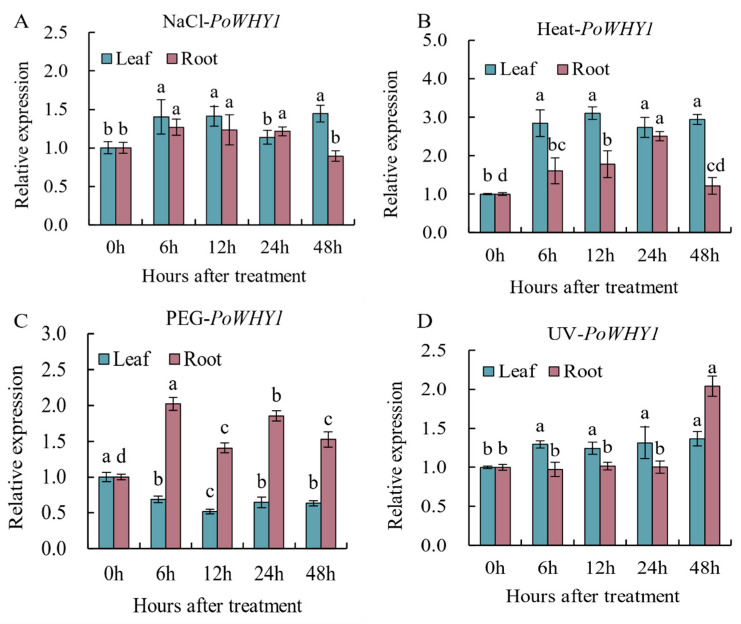
Expression patterns of *PoWHY1* in response to NaCl, heat, PEG6000, and UV-C stresses. The different conditions are not being directly compared statistically. (**A**) shows the *PoWHY1* expression level under salt stress, (**B**) displays the *PoWHY1* expression level under heat stress, (**C**) illustrates the *PoWHY1* expression level under PEG stress, and (**D**) represents the *PoWHY1* expression level under UV stress. The letters a–d indicate the statistical significance between the different groups. Groups sharing the same letter (e.g., a) are not significantly different (*p* ≥ 0.05), while groups marked with different letters (e.g., a vs. b) are significantly different (*p* < 0.05).

During growth, *P. orientalis* faces complex stresses such as high salinity, drought, etc., and its living environment is not always suitable. To determine whether *PoWHY1* is involved in the response of *P. orientalis* to abiotic stress, qRT-PCR was used to analyze the expression changes of *PoWHY1* gene under high salinity, high temperature, and PEG6000 and UV-C stresses. The results are shown in Figure 4. Exposure to 300 mmol/L NaCl induced varying levels of *PoWHY1* gene expression in the leaves of *P. orientalis*, with higher relative expression observed at 6 h, 12 h, and 48 h compared to untreated leaves. The expression level of *PoWHY1* gene in the roots first increased and then decreased, and the expression of *PoWHY1* gene was upregulated to 1.27 times the value at 0 h after 6 h. The expression level of *PoWHY1* in leaves increased greatly under high-temperature stress at 40 °C, showing a 2.73- to 3.10-fold increase compared to 0 h, and there was no significant difference between the 6~48 h treatment. As the treatment time increased, the expression of *PoWHY1* in roots first increased and then decreased, reaching the highest value at 24 h, which was 2.51 times higher than that at 0 h. In addition, 15% PEG6000 stress also affected *PoWHY1* expression, and the *PoWHY1* gene expression trend was reversed in roots and leaves. The relative expression of *PoWHY1* gene in leaves was downregulated to the lowest value at 12 h, which was 0.52 times higher than that at 0 h, while the highest expression in roots was 2.02 times higher at 0 h. UV-C induced the expression of *PoWHY1*, and the expression level of *PoWHY1* gene in roots did not change significantly during the 0~24 h treatment but increased to 2.04-fold after 48 h upregulation, while *PoWHY1* was upregulated to different extents in the leaves at different treatment times, reaching the highest value after 48 h.

### 2.5. ABA Regulates PoWHY1 Expression in P. orientalis

**Figure 5:**
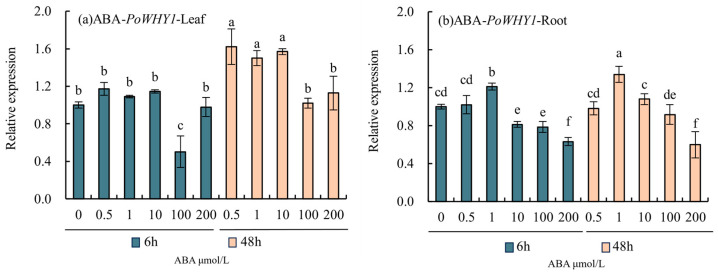
Effects of ABA on the expression levels of *PoWHY1* under normal conditions. The letters a–f represent statistical significance between the groups. Groups labeled with the same letter (e.g., a) are not significantly different from each other (*p* ≥ 0.05), while groups marked with different letters (e.g., a vs. b, a vs. c) are significantly different (*p* < 0.05).

Topical administration of different concentrations of ABA under normal conditions induced changes in *PoWHY1* gene expression levels, as shown in Figure 5. With the extension of the ABA application time, the *PoWHY1* expression level of the leaves treated at 48 h was higher than that of the leaves at 6 h, and the expression level of *PoWHY1* reached the highest levels at 6 and 48 h after treatment with 0.5 μmol/L ABA, which were 1.17 and 1.62 times higher than that at 0 h, respectively. Different ABA concentrations caused different changes in the expression of *PoWHY1* in roots, and the expression level of *PoWHY1* was upregulated to the maximum value at 1 μmol/L ABA, which was 1.21 and 1.34 times higher at 6 and 48 h than that at 0 h.

### 2.6. Screening and Analysis of A. thaliana-Positive Plants Transgenic with PoWHY1 Gene

**Figure 6:**
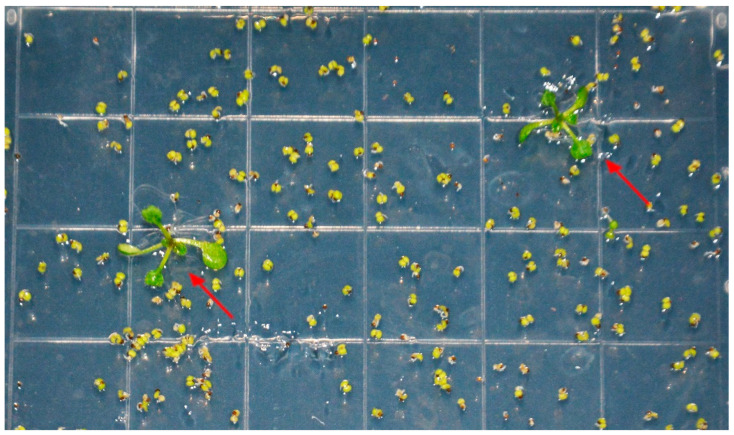
Screening of transgenic *Arabidopsis*-positive plants. The red arrow indicates the positive transgenic line.

The *PoWHY1* gene expression vector was constructed for Agrobacterium transformation, and the inflorescence infection method was used to transform *A. thaliana.* The plant expression vector pCAMBIA1304, containing the HYG resistance marker, was employed for screening. Positive homozygous transgenic *A. thaliana* lines were identified based on their resistance to HYG. T0 transgenic lines were screened for positive transgenic plants on 1/2 MS plates containing 30 mg/L HYG, as shown in Figure 6. The T1 and T2 generations of transgenic plants were screened using the same method and finalized as homozygous lines.

The genomic DNA of homozygous *PoWHY1* transgenic *A. thaliana* and wild-type *A. thaliana* was extracted from a single plant, and the target gene *PoWHY1* was PCR amplified with specific primers to molecularly identify the *PoWHY1* transgenic lines (Figure S2).

### 2.7. Expression Levels of PoWHY1 in Transgenic Homozygous Lines

**Figure 7:**
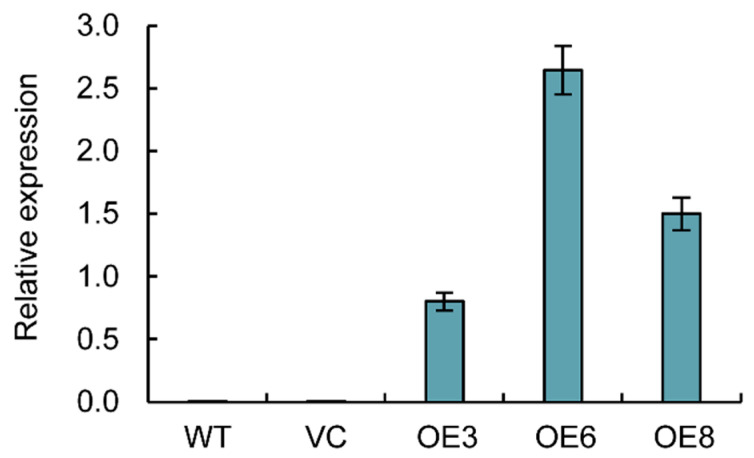
*PoWHY1* expression levels of wild type (WT), vector control (VC), and transgenic lines (OE3, OE6, and OE8).

The total RNA of the homozygous *A. thaliana* transgenic lines that were positive in HYG screening and molecular identification was extracted, and the expression level of the *PoWHY1* gene in the *A. thaliana* transgenic lines was determined by qRT-PCR, which, as well as the expression level of the *PoWHY1* gene detected in the transgenic *PoWHY1* lines OE3, OE6, and OE8, is shown in Figure 7 for further analysis.

### 2.8. Effects of Overexpression of the PoWHY1 Gene on Salt and Drought Stress in A. thaliana

#### 2.8.1. Overexpression of PoWHY1 Gene Increased Seed Germination Rate in *A. thaliana* Under Salt Stress

**Figure 8:**
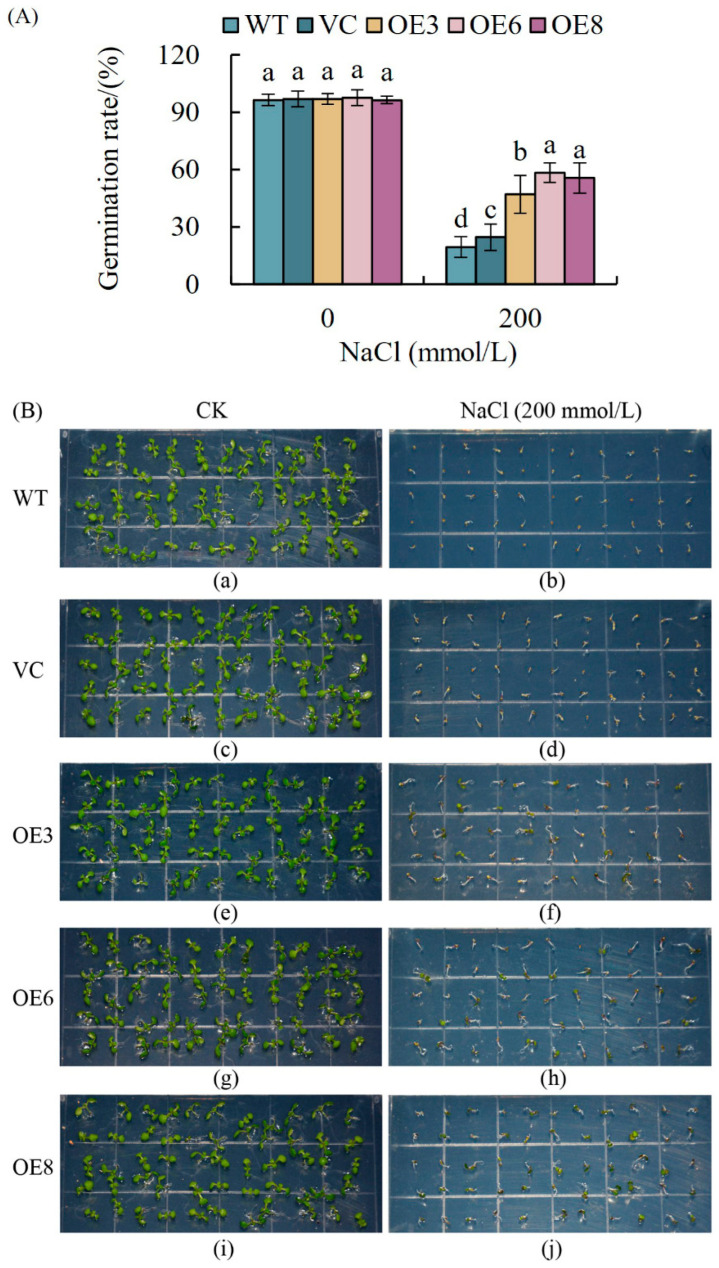
Overexpression of *PoWHY1* enhances the resistance of *A. thaliana* seeds to salt stress. Germination rate (**A**) and growth performance (**B**) of WT, VC, and transgenic lines on 1/2 MS medium supplemented with 0 and 200 mmol/L NaCl. The germination rate was calculated on the 5th day. Photographs were taken on the 9th day after germination. The letters a–d represent statistical significance between the different groups. Groups labeled with the same letter (e.g., a) are not significantly different (*p* ≥ 0.05), while groups marked with different letters (e.g., a vs. b, c vs. d) indicate significant differences (*p* < 0.05).

As shown in Figure 8A, the seed germination rate of *PoWHY1* transgenic *A. thaliana* (OE3, OE6, and OE8) under normal CK (control, untreated group) conditions was close to that of WT and VC, at 96.36% to 97.58%. The germination rate of *PoWHY1* transgenic *A. thaliana* seeds was higher than that of WT and VC, with 47.05%, 58.47%, and 55.69% for OE3, OE6, and OE8 and 19.50% and 24.62% for WT and VC. We sowed these five *A. thaliana* seeds in 1/2 MS medium with 200 mmol/L NaCl each. As shown in Figure 8B, WT and VC growth was significantly inhibited after 9 days of sowing compared to *PoWHY1* transgenic plants, indicating that overexpression of *PoWHY1* increased NaCl resistance in *A. thaliana* seeds.

#### 2.8.2. Overexpression of the *PoWHY1* Gene Increased the Tolerance of *A. thaliana* to Salt Stress

**Figure 9:**
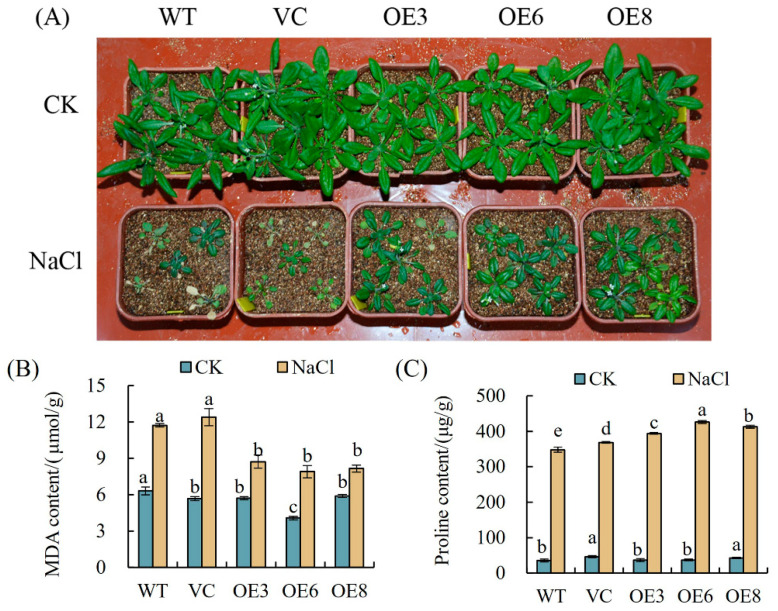
Salt tolerance analyses of WT, VC, and *PoWHY1* transgenic *A. thaliana*. (**A**) The phenotypes of 17-day-old PoWHY1 transgenic Arabidopsis, WT, and VC plants under normal growth conditions (top panel) and the same plants after being transferred to 300 mmol/L NaCl for 15 days (bottom panel). The reduced size and leaf loss observed in the treated plants are indicative of salt stress effects. (**B**,**C**) The MDA and proline contents of the leaves of all plants after high-salt treatment and under normal growth conditions. The different conditions are not being directly compared statistically. The letters a–e represent statistical significance among the groups. Groups labeled with the same letter (e.g., a) are not significantly different (*p* ≥ 0.05), whereas groups with different letters (e.g., a vs. b, a vs. e) show significant differences (*p* < 0.05).

The *PoWHY1*-overexpressing phenotype of the *A. thaliana* line (OE3, OE6, and OE8) was similar to that of WT and VC, but transgenic *PoWHY1 A. thaliana* under high-salt conditions delayed the overall aging process. As shown in Figure 9A, leaf wilting was more severe in WT and VC plants than in transgenic lines (OE3, OE6, and OE8) after 15 days of salt stress. The MDA content in the leaves of transgenic plants was significantly lower than that of WT and VC under salt stress (Figure 9B). In addition, the changes in leaf proline content caused by salt stress are shown in Figure 9C. The proline content of WT, VC, and transgenic plants increased after 15 days of salt stress, and the proline content of WT and VC plants was 348.17 μg/g and 368.71 μg/g, respectively, while the proline content of transgenic plants significantly increased by 394.08–426.76 μg/g. These results tentatively suggest that overexpression of *PoWHY1* increases salt resistance in *A. thaliana*.

**Figure 10:**
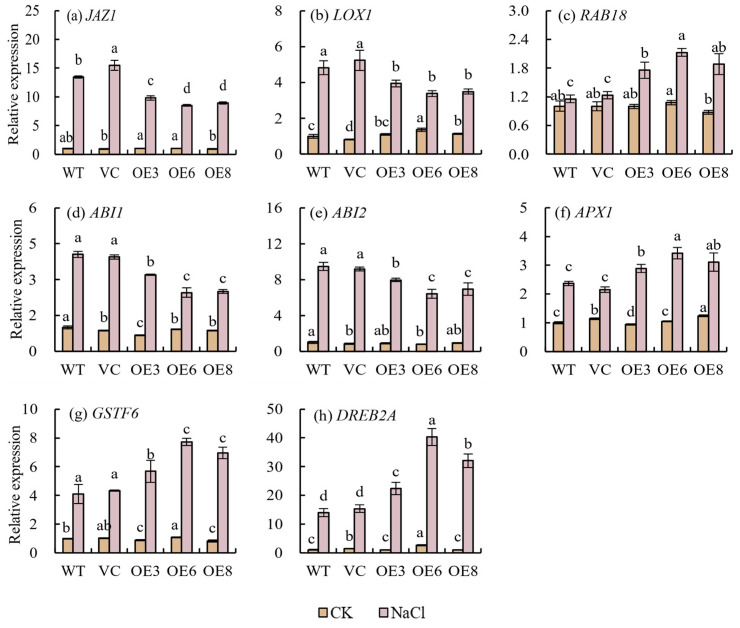
Expression of stress-related genes in WT, VC, and transgenic lines in response to NaCl stress. The different conditions are not being directly compared statistically. The letters a–d indicate statistical significance among the groups. Groups with the same letter (e.g., a) are not significantly different (*p* ≥ 0.05), while groups with different letters (e.g., a vs. b, c vs. d) are significantly different (*p* < 0.05).

To further investigate the role of *PoWHY1* in salt stress and investigate its possible molecular mechanism in plants, the transcriptional response of known stress-related genes caused by the overexpression of *PoWHY1* was examined by qRT-PCR (Figure 10). These genes include JA response genes (*JAZ1* and *LOX1*), genes related to the ABA signaling pathway (*ABI1*, *ABI2*, and *RAB18*), genes related to reactive oxygen species scavenging (*APX1* and *GSTF6*), and dehydration-inducing genes (*DREB1A* and *DREB2A*). As shown in Figure 10, under normal conditions, there was no significant difference in the transcriptional expression of *PoWHY1* transgenic seedlings and most of the detected genes in WT and VC. After 15 days of salt stress treatment, the transcription levels of *JAZ1* and *LOX1* genes in the transgenic lines were significantly lower than in WT and VC plants. Furthermore, salt stress caused different increases in the expression levels of *ABI1*, *ABI2*, and *RAB18* in WT, VC, and transgenic plants, but the expression levels of ABI1 and ABI2 in transgenic plants were significantly lower than those in WT and VC, while *RAB18* was the opposite. Overexpression of *PoWHY1* significantly increased the transcription levels of *APX1* and *GSTF6* genes. Similarly, the overexpression of *PoWHY1* also increased the expression level of *DREB2A* gene under salt stress, and the expression of *DREB2A* in WT and VC plants was upregulated under salt stress, and this upregulation trend was greatly enhanced by the overexpression of *PoWHY1*. The above analysis also suggests that *PoWHY1* may play an important role in the salt stress response pathway.

#### 2.8.3. Overexpression of the *PoWHY1* Gene Increases Drought Stress Tolerance of *A. thaliana*

**Figure 11:**
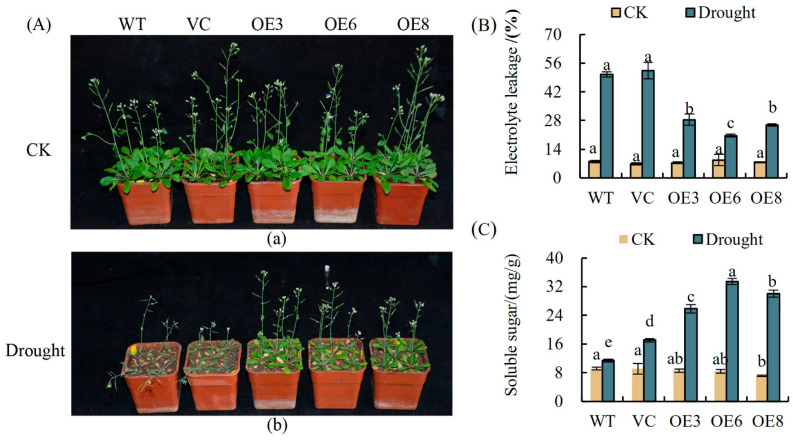
Drought tolerance analyses of transgenic *A. thaliana* seedlings under drought stress conditions. (**A**) Two-week-old transgenic, WT, and VC plants were transplanted to soil for an additional one week and then subjected to drought for 18 days; photographs of representative seedlings were taken. (**B**,**C**) Electrolyte leakage and soluble sugar contents in transgenic, WT, and VC plants at 14 days under drought stress. The different conditions are not being directly compared statistically. (a) and (b) represent the experimental group under Control check and drought stress respectively. The letters a–e represent statistical significance among the groups. Groups with the same letter (e.g., a) are not significantly different (*p* ≥ 0.05), while groups with different letters (e.g., a vs. b, a vs. e) indicate significant differences (*p* < 0.05).

As shown in Figure 11A, the three-week-old transgenic plants showed strong drought resistance compared to WT and VC plants after 18 days of irrigation, while the WT and VC plants were severely desiccated and withered. To evaluate the physiological changes between *PoWHY1* transgenic plants and WT and VC under drought stress, the electrolyte extravasation rate and soluble sugar content were determined. Under normal CK conditions, the electrolyte extravasation rate and soluble sugar content in the leaves of transgenic plants were similar to those in WT and VC leaves. The electrolyte extravasation rates of OE3, OE6, and OE8 were 28.41%, 20.57%, and 25.83%, respectively, which were significantly lower than those of WT and VC *A. thaliana* leaves, which were 50.53% and 52.43%, respectively (Figure 11B). Drought stress resulted in varying degrees of increase in the soluble sugar content of WT, VC, and transgenic leaves, and compared to WT and VC plants, the soluble sugar content increased more in the leaves of transgenic plants (Figure 11C). It was concluded that overexpression of *PoWHY1* increased the drought resistance of *A. thaliana*.

**Figure 12:**
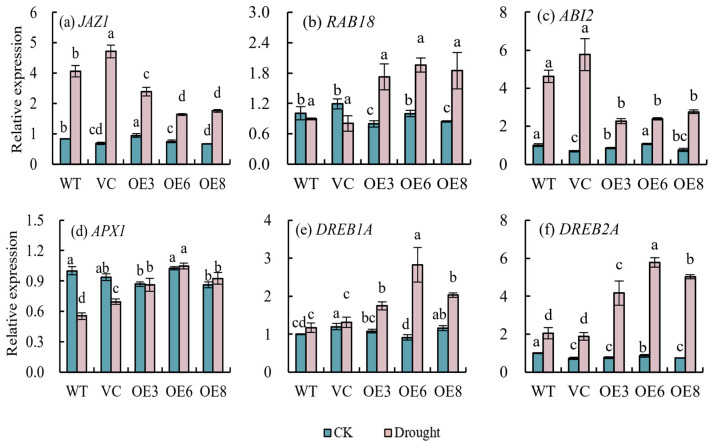
Expression of stress-related genes in WT, VC, and transgenic lines in response to drought stress. The different conditions are not being directly compared statistically. The letters a–d represent statistical significance between the different groups. Groups labeled with the same letter (e.g., a) are not significantly different (*p* ≥ 0.05), while groups marked with different letters (e.g., a vs. b, c vs. d) indicate significant differences (*p* < 0.05).

At the same time, as shown in Figure 12, the expression level of the *JAZ1* gene in transgenic plants after 14 days of drought stress was significantly lower than that of WT and VC. Furthermore, there were significant differences between WT, VC, and transgenic plants under drought stress, and the transcription level of the *APX1* gene in transgenic plants was significantly higher than that in WT and VC plants. At the same time, drought stress caused significant upregulation of the *ABI2* gene, and the expression level of *ABI2* in transgenic plants was significantly lower than that of WT and VC, while the expression level of the *RAB18* gene in transgenic plants was significantly higher than that of WT and VC. Furthermore, overexpression of *PoWHY1* increased the expression levels of *DREB1A* and *DREB2A* under drought stress compared to WT and VC plants.

## 3. Discussion

With environmental degradation and continuous population growth, the effects of abiotic stress on plant growth and development are becoming more and more serious, which in turn poses a serious threat to food security, the ecological environment, and the economy [37]. The molecular response of plants to these adverse environmental factors is complex, and the abiotic stress process involves multistep responses such as stress sensing, signal transduction, transcription, transcriptional processing, translation, and posttranslational protein modification [38,39]. Among them, transcription factors play a very important role in plant defense responses and stress responses [40,41]. This study demonstrated that the expression of the *PoWHY1* gene was significantly upregulated under salt and drought stresses, and its overexpression effectively enhanced salt and drought tolerance in *Arabidopsis thaliana*. These findings highlight the central role of *PoWHY1* as a key transcription factor, consistent with studies on *WHY1* genes in other plants [42,43]. For example, overexpression of the *SlWHY1* and *SlWHY2* gene in tomato improved both drought and salt tolerance [13], while in barley, *TaWHY* delayed leaf senescence by regulating the expression of the senescence-associated gene *HvS40* [44].

*WHY1*, located in the plastids of *A. thaliana*, can respond to exogenous ABA [45] and is an important upstream regulator of drought stress response [46]. *WHY1* influences DNA structure through its interaction with DNA (plastid and nucleus) and also with genes associated with aging stress [47]. *P. orientalis* will experience various abiotic stresses during the growth cycle, and the extremely harsh environment affects the quality of *P. orientalis*, thereby reducing the economic and environmental benefits of *P. orientalis* [48,49]. The molecular mechanism of *WHY* is well elucidated in a variety of plants, but the physiological function and molecular regulation mechanism of the *WHY1* gene under abiotic stress in woody plants are still poorly understood. This study revealed the critical role of the *PoWHY1* gene from *Platycladus orientalis* under various abiotic stresses, including drought, salinity, UV-C, and ABA treatment. Its function in stress responses was validated using transgenic *Arabidopsis thaliana* as a model, providing new insights into the regulatory mechanisms of *PoWHY1*.

As key regulators of gene expression, transcription factors play a crucial role in coordinating abiotic stress responses and age-related signaling pathways through gene regulation [50]. In various plant species, specific transcription factors are components of stress-induced regulatory pathways of leaf senescence [5,51,52]. Previous studies have shown that *AtWHY1* inhibits *WRKY53* expression and delays leaf senescence depending on the developmental stage by binding its promoter-like ERE-like elements and AT enrichment sequences in *A. thaliana* during leaf senescence [28]. This study found that transgenic *Arabidopsis thaliana* plants overexpressing *PoWHY1* exhibited significantly enhanced tolerance to salt and drought stresses. These plants showed reduced malondialdehyde (MDA) content, increased accumulation of proline and soluble sugars, and decreased electrolyte leakage, indicating that *PoWHY1* alleviates stress effects by reducing oxidative damage and enhancing osmotic regulation. Gene expression analysis revealed the upregulation of key stress-responsive genes, such as *RAB18*, *DREB2A*, and *APX1*, along with the downregulation of negative regulators like *ABI1* and *JAZ1*. These findings suggest that *PoWHY1* coordinates ABA- and JA-mediated signaling pathways to enhance stress tolerance. Additionally, under heat stress, the expression of *PoWHY1* was significantly upregulated in *Platycladus orientalis*, suggesting its potential role in thermotolerance. Transgenic *A. thaliana* plants overexpressing *PoWHY1* exhibited delayed heat-induced leaf senescence, accompanied by the upregulation of heat shock protein genes such as *HSP70* and *HSP101*. This indicates that *PoWHY1* may regulate heat stress signaling pathways by maintaining protein homeostasis and mitigating cellular damage. Under UV stress, UV-C treatment significantly induced the expression of *PoWHY1* in *P. orientalis*. In transgenic *A. thaliana* plants, UV-C stress activated the antioxidant enzyme system, including increased expression levels of ascorbate peroxidase (APX) and superoxide dismutase (SOD). These results suggest that *PoWHY1* protects cellular structures from oxidative damage by scavenging reactive oxygen species (ROS), thereby maintaining cellular integrity.

During growth, plants respond to stress with a variety of endogenous hormones and allocate plant resources between growth, development and defense to improve survival under adverse conditions [53,54,55]. As a key stress hormone, abscisic acid (ABA) plays a critical role in coordinating plant responses to abiotic stresses [56,57,58]. ABA treatment significantly induced the expression of *PoWHY1* in *Platycladus orientalis*, suggesting its function as a positive regulator in stress response pathways. In transgenic *Arabidopsis thaliana* plants, *PoWHY1* enhanced the expression of ABA-responsive genes, such as *RAB18* and *RD29B*, indicating that *PoWHY1* may promote stress adaptation by amplifying ABA signaling. Furthermore, the downregulation of *ABI1* provides additional evidence that *PoWHY1* fine-tunes ABA-dependent pathways to improve stress tolerance.

Although this study revealed the function of *PoWHY1* in *Arabidopsis thaliana*, its specific molecular mechanisms in *Platycladus orientalis* require further investigation. For instance, it remains unclear whether *PoWHY1* directly binds to specific promoters to regulate the expression of stress-related genes. Additionally, whether the findings in *A. thaliana* are fully applicable to *P. orientalis* needs to be validated through further studies, such as CRISPR/Cas9-based functional analysis. Future research should explore the role of *PoWHY1* under natural stress conditions through more comprehensive functional validation experiments.

## 4. Materials and Methods

### 4.1. RNA Extraction and cDNA Synthesis

Total RNA was extracted from the leaves of 500-year-old *P. orientalis* trees using the column plant RNA isolation kit reagent and stored in a freezer at −80 °C. For reverse transcription synthesis of the first strand of cDNA, see the SMARTer™ RACE cDNA Amplification Kit. (Clontech Laboratories, Inc., Mountain View, CA, USA).

### 4.2. Gene Cloning and Bioinformatic Analysis

The *PoWHY1* gene sequence was obtained from the NCBI database, and primers were designed using Primer Premier 6.0 to amplify the full-length cDNA sequences of *PoWHY1-GSP1* and *PoWHY1-GSP*, respectively (Table S1). The 5′ and 3′ ends of the *PoWHY1* cDNA were amplified using the first cDNA strand as a template for RACE-PCR. PCR reactions (50 μL) were as follows: 5′-RACE: 2.5 μL cDNA, 5 μL UPM (10×), 1 μL *GSP1* (10 μm), and 41.5 μL master mix; 3′-RACE: 2.5 μL cDNA, 5 μL UPM (10×), 1 μL *GSP2* (10 μm) and 41.5 μL Master Mix. To detect the amplified fragments, 1.0% agarose gel electrophoresis was performed, the obtained products were ligated to the *pMD-19* cloning vector, the ligation products were transferred to *DH5α*-competent cells, the plates were coated, and the white single colonies were prepared for PCR selected. Positive clones were selected and sent to the biotechnology company for sequencing.

We carried out analysis of full-length cDNA sequence of *PoWHY1* using ORF Finder; we conducted amino acid sequence and domain analysis of *PoWHY1* using NCBI (https://www.ncbi.nlm.nih.gov/Structure/cdd/wrpsb.cgi) (accessed on 15 August 2023); we carried out prediction of isoelectric point and molecular weight of *PoWHY1* using ProtParam (https://web.expasy.org/protparam/)(accessed on 15 August 2023); signal peptide prediction was performed using SignalP (http://www.cbs.dtu.dk/services/SignalP/)(accessed on 15 August 2023); secondary structure prediction was performed using HNN (https://npsa-prabi.ibcp.fr/cgi-bin/npsa_automat.pl?page=/NPSA/npsa_hnn.html) (accessed on 15 August 2023); subcellular localization was performed with Wolf PSORT (http://www.genscript.com/wolf-psort.html) (accessed on 15 August 2023); the search for homologous *WHY1* amino acid sequences from other plants in the GenBank database was carried out using NCBI-BLAST, and we performed sequence alignment and phylogenetic tree construction using Genedoc 2.7 and MEGA 60 software.

### 4.3. PoWHY1 Gene Expression Analysis of P. orientalis

Four-month-old seedlings of *P. orientalis* were selected to be cleaned and cultured in plastic containers with 1/4 Hoagland solution for 14 days. Once the seedlings returned to normal growth, they were placed in centrifuge tubes with 1/4 Hoagland’s solution and subjected to various stress treatments. High-salinity and drought stress treatments: four-month-old seedlings were transferred to a 1/4 Hoagland solution containing 300 mmol/L NaCl or 15% PEG6000 for 48 h, respectively [59,60]; high-temperature and UV stress treatments: seedlings were maintained at 40 °C for 48 h or irradiated with UV-C (20 W; wavelength range of 200–275 nm), respectively [61,62]. Leaf and root samples were collected at 0, 6, 12, 24, and 48 h, respectively. In addition, different concentrations of ABA (0, 0.5, 1, 10, 100, and 200 μmol/L) were applied to *P. orientalis* grown under normal conditions and samples were stored at −80 °C for RNA extraction and further analyses were undertaken.

Three 20-year-old *P. orientalis* trees were selected for gene expression analysis in roots, leaves, stems, seeds, and fruits. CDNA was obtained by reverse transcription of *P. orientalis* RNA, and *αTUB* was used as an internal reference gene of *P. orientalis* to analyze the expression of *PoWHY1* gene by quantitative fluorescence PCR (qRT-PCR).

### 4.4. Vector Construction and Screening of Transgene-Positive Plants

**Figure 13:**
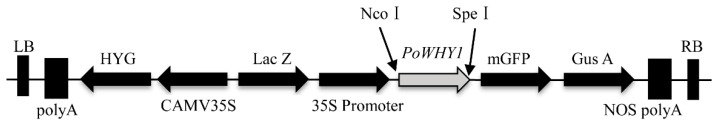
Schematic diagram of the *pCAMBIA1304-PoWHY1*.

The open reading frame (ORF) of *PoWHY1* was amplified using the cDNA of the gene of interest *PoWHY1* with primers containing NcoI. and SpeI. digestion sites (Table S1) using the high-fidelity enzyme PrimeSTAR. The amplified product was recovered and ligated to the pMD19-T vector to transform *E. coli* DH5α competent cells. The transformed bacterial solution was tested for Amp resistance and cultured overnight, and then a single colony was picked and the positive plasmid was identified by PCR and enzyme digestion. The cloned plasmid with the correct sequence was digested with NcoI. and SpeI., and the plasmid of pCAMBIA1304 expression vector was digested with these two enzymes. The recovered digested products were ligated with T4 DNA ligase to construct the plant expression vector *pCAMBIA1304-PoWHY1*, as shown in Figure 13. The ligation product was transformed into *E. coli* DH5α competent cells and the transformed bacterial solution was screened for Kan resistance and identified by PCR; positive clones were selected, and the plasmid was extracted for NcoI. and SpeI. Double Digestion Review. Single colonies of Agrobacterium tumefaciens GV3101 were selected and placed in LB liquid medium to produce competent Agrobacterium tumefaciens cells. Agrobacterium-competent cells were transformed into Agrobacterium-competent cells by electroshock to the recombinant plasmid *pCAMBIA1304-PoWHY1* or unloaded plasmids pCAMBIA1304, respectively, and then inflorescence infection was used for *A. thaliana* transformation. (Please refer to the *A. thaliana* transformation protocol available here: (https://blog.addgene.org/tips-for-arabidopsis-transformation, accessed on 15 August 2023). DNA from *A. thaliana* was extracted using the CTAB method, and PCR-positive lines were detected using specific primers for the target gene. Finally, homozygous transgenic positive lines were identified.

### 4.5. Detection of A. thaliana Gene Expression Level of Transgenic PoWHY1 Gene

To better understand the expression of exogenous genes in transgenic plants, gene expression detection was carried out on transgenic lines. *A. thaliana* total RNA was extracted by Trizol extraction followed by reverse transcription to obtain cDNA, and *Actin1* (NM_179953.2) was used as an internal reference gene while qRT-PCR analyzed gene expression.

### 4.6. Analysis of A. thaliana Tolerance to Abiotic Stresses in the PoWHY1 Gene

To demonstrate the stress resistance of *A. thaliana* lines overexpressing *PoWHY1* gene, surface-sterilized transgenic T3 lines and wild-type (WT) and empty vector (VC) seeds were cultured on 1/2 MS medium, and the relevant physiological indices (MDA, external electrolyte assessment rate, proline content, and soluble sugar content) were measured and gene expression analysis was performed. Salt stress test: 10-day-old seedlings were transferred from the medium to 1/2 MS nutrient solution (pH 6.0) for about 7 days and then to 1/2 MS nutrient solution containing 300 mmol/L NaCl for 15 days. Drought stress test: 14-day-old seedlings were transferred to nutrient medium (medium: vermiculite:perlite = 3:1:1) for about 7 days and then watering was stopped until an obvious difference was seen.

### 4.7. Measurement of Physiological Index

(1) Determination of malondialdehyde (MDA): MDA in fresh leaves was extracted in 10% trichloroacetic acid and centrifuged, and the supernatant was mixed with 0.67% TBA, heated in boiling water for 30 min, and cooled and centrifuged, and the absorption of the supernatant was measured at different wavelengths (450, 532, and 600 nm) and the MDA content was calculated [63].
(2) Determination of electrolyte extravasation: Fresh leaves were washed with deionized water, dried, and kept in deionized water at room temperature for 24 h, and the relative conductivity of the solution was determined with a conductivity meter (DDS-11A) EC1. The boiling water was then heated for 15 min, and the cooled solution had an electrical conductivity of EC2 to calculate the relative conductivity [64].
(3) Determination of proline content: 3% sulfosalicylic acid was added to fresh leaves, heated in boiling water for 10 min, and filtered to obtain the extract. The extract, glacial acetic acid, and acid ninhydrin were taken in a ratio of 1:1:1, mixed in boiling water for 30 min, and cooled, then toluene was added, the mixture was centrifuged with the upper liquid, and the absorbance value was measured at 520 nm and the content calculated according to the standard curve [65].
(4) Determination of soluble sugar content: The leaves were ground in liquid nitrogen, then distilled water was added and boiled. The supernatant was removed and anthone ethyl acetate reagent and concentrated sulfuric acid were added, mixed well, and heated in boiling water for 1 min. After cooling, the absorbance was measured at 630 nm and the soluble sugar content was calculated according to the standard curve [66].

### 4.8. Seed Germination Experiment of PoWHY1 Transgenic A. thaliana Under NaCl Treatment

*A. thaliana* seeds of the WT, VC, and *PoWHY1* transgenic lines (OE3, OE6, and OE8) were sterilized and sown on 1/2 MS medium with 0 and 200 mmol/L NaCl, respectively. Germination rates were calculated on the 5th day.

### 4.9. Expression Analysis of Genes Related to Transgenic A. thaliana

We extracted total RNA from *A. thaliana* then performed reverse transcription and qRT-PCR using cDNA as a template, with *A. thaliana* Actin1 as a reference gene. The GenBank accession numbers for stress-related genes are as follows: *JAZ1* (NM_1017762), *LOX1* (NM_1043762), *ABI1* (NM_1187413), *ABI2* (Y089651), *RAB18* (U756031), *GSTF6* (NM_0011979641), *APX1* (NM_0011237721), *DREB1A* (AB0077871), *DREB2A* (AB0077901), etc. (Table S2).

### 4.10. Statistical Analysis

All statistical analyses were performed with SPSS Statistics 19.0 by one-way ANOVA. Values are expressed as the means ± SD of three replications. Statistical significance was reported at *p* < 0.05.

## 5. Conclusions

In this study, the *PoWHY1* gene from *Platycladus orientalis* was cloned and functionally characterized. The results showed that *PoWHY1* expression was significantly induced under abiotic stresses, including high salinity, drought, heat, UV-C, and ABA treatment. Overexpression of *PoWHY1* in *Arabidopsis thaliana* enhanced tolerance to salt and drought stresses, as evidenced by reduced malondialdehyde (MDA) accumulation, increased proline and soluble sugar levels, and decreased electrolyte leakage. Furthermore, *PoWHY1* positively regulated the expression of key stress-related genes, such as *RAB18*, *DREB2A*, and *APX1*, while downregulating negative regulators like *JAZ1* and *ABI1*, suggesting its involvement in coordinating ABA- and JA-mediated stress response pathways. These findings demonstrate that *PoWHY1* plays a critical role in enhancing plant tolerance to abiotic stresses and provide a foundation for future functional studies of the *WHY* gene family in *P. orientalis*.

## Data Availability

The original contributions presented in this study are included in the article/Supplementary Materials. Further inquiries can be directed to the corresponding authors.

## Supplementary Materials

The following supporting information can be downloaded at: https://www.mdpi.com/article/10.3390/plants14020218/s1.
