# Supplementary material for: Functional Characterization of the PoWHY1 Gene from Platycladus orientalis and Its Role in Abiotic Stress Tolerance in Transgenic Arabidopsis thaliana
Source: Plants (Basel). 2025 Jan 14;14(2):218. doi: 10.3390/plants14020218 (PMC11768397; doi:10.3390/plants14020218)
Supplement: Supplementary file 1 [file plants-14-00218-s001.zip › plants-3346612-supplementary.pdf]

supplemental material

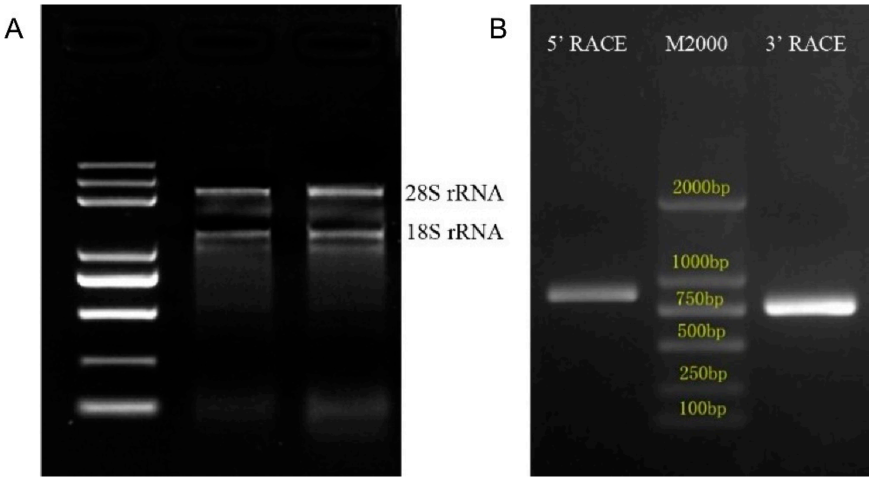

Note: (A) Electrophoretogram of total RNA from *P. orientalis*; (B) Products of 3′ RACE and 5′ RACE

Figure S1. Agarose gel electrophoresis analysis of the *PoWHY1* gene in *P. orientalis*

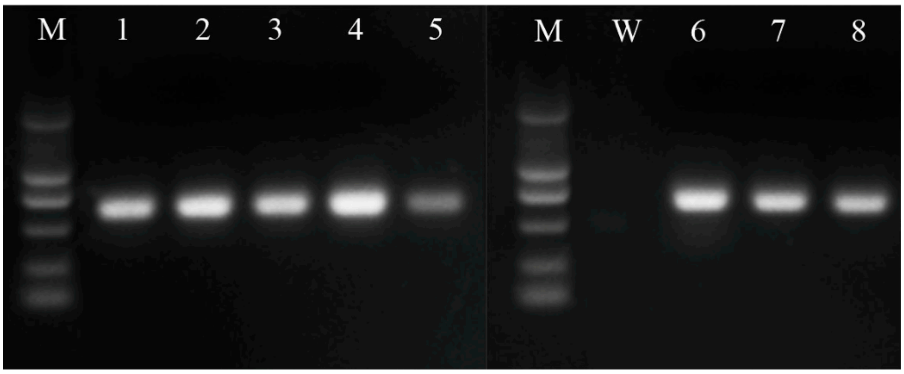

Note: M: DNA ladder marker 2000; 1-8: *PoWHY1* transgenic lines; W: Wild-type *A. thaliana*.

Figure S2. PCR analysis of transgenic lines overexpressing *PoWHY1*

Table S1 *PoWHY1* gene cloning and related gene primers

Primer name	Primer sequence (5’→3’)	Primer sequence strategy
PoWHY1-GSP1	CTGATGTTCTCCGATATTGACAGAGTT	5′ RACE Gene-specific primers for 5′RACE
PoWHY1-GSP2	TTACAGTGATGCGGTCCTCTTTC	3′ RACE Gene-specific primers for 3′RACE
Universal Primer A Mix	CTAATACGACTCACTATAGGGC	5'- & 3'-RACE PCR
PoWHY1-ORF1	ATGTCAAAGGCGCTCAAGCTAGTT	ORF amplification of *PoWHY1*
PoWHY1-ORF2	TTATGATGATACTTGCTGATGTTCTCCG	ORF amplification of *PoWHY1*
PoWHY1-OF	CCATGGATGTCAAAGGCGCTCAAGCTA GTT	Overexpression vector construction
PoWHY1-OR	ACTAGTTGATGATACTTGCTGATGTTCT CCG	Overexpression vector construction
PoWHY1-qF	GAGCTTCTCCCGTTTCCAGG	qRT-PCR
PoWHY1-qR	GGGCAGAATTCCGGTGCTAT	qRT-PCR

Note: CCATGG and ACTAGT underlined in primers indicate restriction enzyme sites of *NcoI* and *SpeI*

Table S2 The *A. thaliana*-related genes primers used for qRT-PCR

Gene Name	Accession no.	Primer sequence (5'→3')
JAZ1	NM_101776.2	GCCAATCCAATCCTCCCCAA (Forward)<br>GGGTTTGAAGACGCTTTGGC (Reverse)
LOX1	NM_104376.2	TAGGCGTCCCACCAAAGAAC (Forward)<br>GTGATCGGTAGCCTGCTCTC (Reverse)
ABI1	NM_118741.3	TGGCGGTTCTCAGGTAGCG (Forward)<br>TCCAGCCACGTATCACCATC (Reverse)
ABI2	Y08965.1	ACTTCAGTGCGGCGAGTAAA (Forward)<br>TTCTCCTCTTTTCTCCGCCG (Reverse)
RAB18	U75603.1	TTTGCTCGGGAGTACGGATG (Forward)<br>CTGTGCGGGGTTTTGTTTGA (Reverse)
GSTF6	NM_001197964.1	AACACAGGCTTGGTGAGTCC (Forward)<br>GCAACCCAAGCACTCACATG (Reverse)
APX1	NM_001123772.1	GGACGATGCCACAAGGATAGG (Forward)<br>GGAAAACAGGGTCGTCCAATAGT (Reverse)
DREB1A	AB007787.1	GCCGATCAGCCTGTCTCAAT (Forward)<br>TCCGCCGTGTAAATAGCCTC (Reverse)
DREB2A	AB007790.1	AAACCTGTCAGCAACAACAGCAGG (Forward)<br>TTAAGCCTGCAAACACATCGTCGC (Reverse)
